# Comment on “Humic acid-dependent respiratory growth of *Methanosarcina acetivorans* involves pyrroloquinoline quinone” by Yuanxu Song *et al*.

**DOI:** 10.1093/ismejo/wrae020

**Published:** 2024-02-02

**Authors:** Derek R Lovley, Dawn E Holmes

**Affiliations:** Department of Microbiology, University of Massachusetts-Amherst, 639 North Pleasant Street, Amherst, MA 01003, United States; Department of Microbiology, University of Massachusetts-Amherst, 639 North Pleasant Street, Amherst, MA 01003, United States; Department of Physical and Biological Science, Western New England University, 1215 Wilbraham Road, Springfield, MA 01119, United States

**Keywords:** extracellular electron transfer, multi-heme cytochrome, Methanosarcina, humic acids

Song *et al*. report that they have found a novel mechanism for extracellular electron transfer during “humic acid-dependent respiratory growth of *Methanosarcina acetivorans*” [1]. However, as detailed below, an analysis of their study reveals that electron transfer to humic substances accounted for <0.5% of the electron flux during *M. acetivorans* growth. More than 99.5% of electron flux was directed toward methane production. Therefore, the mutant phenotype and transcriptome patterns that Song *et al*. attributed to electron transfer to humic substances have little relevance to humic acid reduction. Their mutant study also failed to directly examine the humics reduction phenotype.

Previous studies have demonstrated that *M. acetivorans* grew with reduction of the humic acid analog anthraquinone-2,6-disulfonate (AQDS) as the sole electron acceptor [2]. AQDS is typically substituted for humic acids in initial investigations on the potential for humic acids reduction because: (1) the quinone moieties of AQDS mimic the quinone moieties that microbes reduce in humic acids and (2) microbes that reduce AQDS have invariably been found to transfer electrons to humic acids [3, 4]. In those previous studies, *M. acetivorans* reduction of AQDS to anthrahydroquinone-2,6-sulfonate was quantitatively documented [2]. Deleting the gene for the outer-surface *c*-type cytochrome membrane multiheme cytochrome A (MmcA) inhibited AQDS reduction. This result suggested that MmcA was an important component of extracellular electron transport to AQDS (and by inference humic acids), similar to the multi-heme cytochrome-based extracellular electron transfer in *Shewanella* and *Geobacter* species [5, 6]. Subsequent functional genetic studies have suggested that MmcA is also important in extracellular electron exchange for direct interspecies electron transfer [7] and electrobiocorrosion of iron-containing metals [8].

Song *et al*. state that MmcA is not involved in *M. acetivorans* reduction of humic acids [1]. However, in studies with an MmcA-deficient mutant (which was provided by our lab), the extent of humic acid reduction was not directly compared with the parental strain. Rather, Song *et al*. made their claim based on indirect studies in which parental and MmcA-deficient strains of *M. acetivorans* were grown with methanol as the electron donor in the presence and absence of humic acids. In their comparative studies (Fig. 1), each of the cultures was provided with the same initial concentration of methanol, and all of the methanol was consumed under all conditions. In anaerobic respirations, such as methane production and humic acid reduction, a relatively low proportion (typically 10% or less) of the electron donor is utilized for biosynthesis. Thus, the quantity of electrons available for reduction of terminal electron acceptors was roughly the same in all treatments.

**Figure 1:**
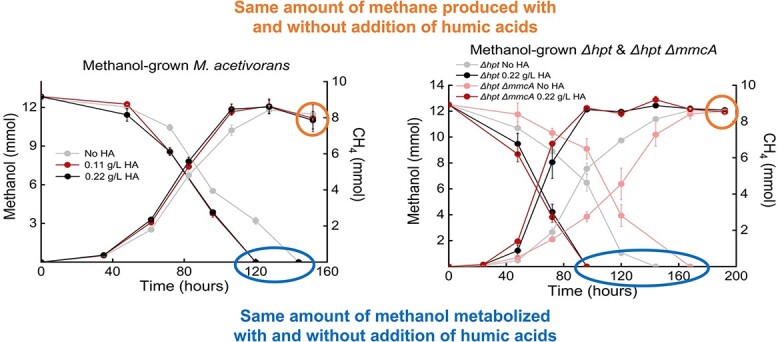
Annotation of data from Song *et al*. [1] to highlight key discussion points in the main text; data plots from panel A (left) and panel E (right) of Song *et al*. [1] Figure 1 are reproduced to emphasize that all cultures metabolized the same amount of methanol and produced the same amount of methane whether or not humic acids were added; studies summarized in the left panel were conducted with the *M. acetivorans* type strain; studies summarized in the right panel were conducted with the *M. acetivorans* parental strain *M. acetivorans* WWM1 (Δ*hpt*) and a strain constructed from strain Δ*hpt* in which the gene for MmcA was deleted, designated strain Δ*hpt*Δ*mmcA*, as previously described [2].

During methanol conversion to methane, methanol is converted to methyl-CoM. One molecule of methyl-CoM is oxidized to carbon dioxide with the recovery of electrons that are then used to reduce three other molecules of methyl-CoM to methane (Fig. 2A). In the absence of extracellular electron acceptors, such as humic acids, *M. acetivorans* can be expected to direct catabolic electron flux solely toward producing methane via methyl-CoM reduction. For generalized illustrative purposes, the quantity of electrons appearing in methane in the absence of an extracellular electron acceptor is designated as M (Fig. 2B and C).

**Figure 2:**
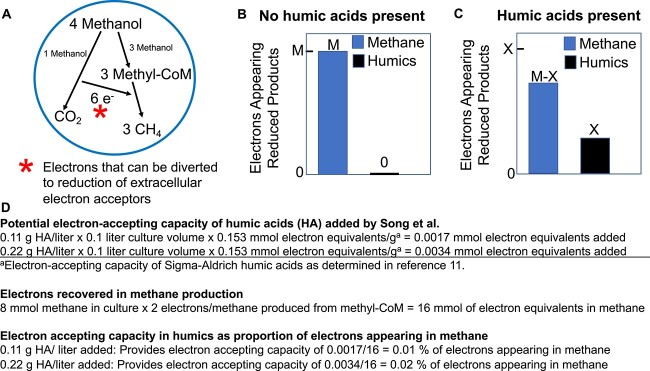
Illustration of expected impacts of adding humic acids as an extracellular electron acceptor to *M. acetivorans* cultures; (A) metabolism of methanol to methane in *M. acetivorans* in which electrons derived from the oxidation of one mole of methanol yields six electrons to support the reduction of three moles of methanol to methane; asterisk designates the electrons that can be diverted to the reduction of an extracellular electron acceptor; (B and C) impact of adding an extracellular electron acceptor on methane production; in the absence of an extracellular electron acceptor, M electron equivalents appear in methane (B); if the same amount of methanol is metabolized in the presence of an extracellular electron acceptor, then the X number of electrons transferred to the extracellular electron acceptor will proportionally decrease the electrons appearing in methane to M-X (C); (D) calculations for determining the electron-accepting capacity of the humic acids added to cultures in Song *et al*. [1] in comparison to the electrons recovered in methane.

In the presence of an extracellular electron acceptor, *M. acetivorans* has two nonexclusive options: (1) continue to route electrons from methanol oxidation to methane production; and/or (2) divert electrons derived from methanol oxidation to reduction of the extracellular electron acceptor. In the presence of the humic acid analog AQDS, *M. acetivorans* simultaneously produced methane and reduced AQDS [2]. For illustration, the quantity of electrons transferred to the extracellular electron acceptor is designated X (Fig. 2B and C).

As noted above, in the studies of Song *et al*., the same amount of methanol was metabolized in all treatments. Thus, the total number of electrons available to support anaerobic respiration was the same in the presence and absence of humic acids. If the electrons appearing in methane in the absence of an alternative electron acceptor is denoted as M, then diversion of X electrons to the reduction of an alternative electron acceptor, such as humic acids, will reduce the quantity of electrons appearing in methane to M-X (Fig. 2B and C). Yet, Song *et al*. report that addition of humic acids had no impact on the total methane produced (Fig. 1). The overlapping endpoints and low standard deviation of the mean suggest that the lack of difference cannot be attributed to sampling errors. This result indicates that humic acids did not significantly divert electron flux away from methane production.

The reason the humic acid amendments had such a small impact is that the electron-accepting capability added in the form of humic acids was extremely low. Humic acids (Sigma-Aldrich) were added at either 0.11 or 0.22 g/l. Sigma-Aldrich humic acids have an electron-accepting capacity of 153 μmoles of electrons/g in physiologically relevant studies [9]. Thus, the electron-accepting capacity of the humic acids added to each 100 ml culture of *M. acetivorans* was 0.0017 or 0.0034 milliequivalents of electrons (see Fig. 2D for calculations). In comparison, the ca. 8 mmoles of methane that accumulated in the presence or absence of humic acids (Fig. 1) represented 16 milliequivalents of electrons (Fig. 2D). Thus, even if *M. acetivorans* had utilized the full physiologically relevant electron-accepting capacity of the humic acids, the humic acids could have accepted at most 0.02% of the electron flux to methane. Electrochemical analyses, which do not have physiological relevance, have indicated that the electron-accepting capacity of some humic acids might be as high as 2.66 mmol/g [10]. Even if this 17-fold higher value is applied, the electron-accepting capacity of the humic acids added to the *M. acetivorans* cultures would have been <0.5% of the electrons recovered in methane. Thus, humics reduction could only have been a trivial path in overall electron flux.

In contrast, previous studies with the humic acid analog AQDS added 32 milliequivalents/l of electron-accepting capacity in the form of AQDS (16 mmoles/l AQDS × 2 electrons accepted/AQDS) [2], which is a 950-fold higher electron accepting capacity per liter than that available from the highest concentration of humic acid that Song *et al*. provided (0.22 g/l × 0.153 mmoles electrons accepted/g = 0.03366 milliequivalents/l). The previous study [2] also added about 10-fold less methanol than Song *et al*. employed. This enabled AQDS to be a major electron acceptor for respiration. Furthermore, when the mechanisms for AQDS reduction were investigated, reduction of methyl-CoM to methane was blocked with the inhibitor 2-bromoethanesulfonate, ensuring that AQDS reduction was the only route for electron flux [2]. It was clear that the MmcA-deficient mutant could not effectively reduce AQDS under these conditions.

The small electron flux to humic acids in the *M. acetivorans* cultures of Song *et al*. means that the phenotype of mutants and transcriptomic patterns cannot be reliably attributed to humic acid reduction mechanisms. Their finding that the MmcA-deficient mutant metabolized methanol in methanol/humic acid media as well as the parental strain is expected because deleting MmcA does not inhibit methanol conversion to methane [2], which was the respiration accounting for over 99.5% of the electron flux in their mutational analysis. Song *et al*. report higher protein and ATP concentrations in cultures amended with humic acids, but the electron transfer to humic acids is much too small to account for these differences, even at the highest conceivable cell yields possible from anaerobic extracellular electron transfer. Thus, the reasons for differences in protein and ATP concentrations, and the transcriptome patterns warrant further study, but cannot be explained by the very small electron flux to humic acids.

Therefore, we do not question the claim that *M. acetivorans* can reduce humic acids because we have previously shown that *M. acetivorans* effectively reduces the humic acid analog AQDS in an MmcA-dependent manner [2]. Our point is that the conclusion by Song *et al*. that MmcA is not involved in electron transfer to humic acids and the inferences from transcriptomic analysis of alternative extracellular electron transfer pathways are not valid. This is because their methane production results, as well as even the most generous assumptions of the electron-accepting capacity of humic acids, demonstrate that humic acid reduction accounted for <0.5% of the electron flux in their humics-amended cultures. Furthermore, Song *et al*. did not conduct the rigorous yet simple test of directly determining whether deleting MmcA reduced the extent of humic acid reduction.

## Conflicts of interest

The authors declare no conflicts of interest.

## Funding

No grant or institutional funding supported the preparation of this manuscript.

## Data availability

Data sharing not applicable to this article as no datasets were generated or analysed during the current study.
